# Early complete response of primary bone marrow B‐cell lymphoma treated with rituximab‐based CHOP therapy, assessed by flow cytometry and immunogloblin heavy chain rearrangement

**DOI:** 10.1002/ccr3.4657

**Published:** 2021-08-16

**Authors:** Yoshimi Nabe, Shohei Kikuchi, Yusuke Kamihara, Akinori Wada, Jun Murakami, Tsutomu Sato

**Affiliations:** ^1^ Department of Hematology Toyama University Hospital Toyama Japan; ^2^ Center for Medical Residency Training Toyama University Hospital Toyama Japan; ^3^ Division of Transfusion Medicine and Cell Therapy Toyama University Hospital Toyama Japan

**Keywords:** IGH rearrangement, primary bone marrow B‐cell lymphoma, rituximab‐based CHOP

## Abstract

Evaluation of early response by flow cytometry and immunogloblin heavy chain assessments against primary bone marrow B‐cell lymphoma could be valuable for predicting treatment outcome.

## Graphical abstract

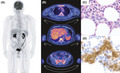

## 1 INTRODUCTION

Primary bone marrow B‐cell lymphoma solely with large B‐cell BM involvement at diagnosis is a rare form of aggressive B‐cell lymphoma, accounted for 1.23% of non‐Hodgkin's lymphoma.^1^ PBML is considered as a distinct subtype of DLBCL with poor prognosis.^1^, ^2^ The use of intensive induction therapies such as R‐Hyper CVAD or R‐CHOP (rituximab, cyclophosphamide, doxorubicin, vincristine, and prednisolone) followed by up‐front high‐dose chemotherapy and autologous stem cell rescue has been reported based on the assumption of poor prognosis.^3^, ^4^ However, because of its rarity, the clinical features of PBML remain unclear and there is no evidence base to support a recommendation of intensive induction therapy.

## 2 CASE HISTORY

**FIGURE 1:**
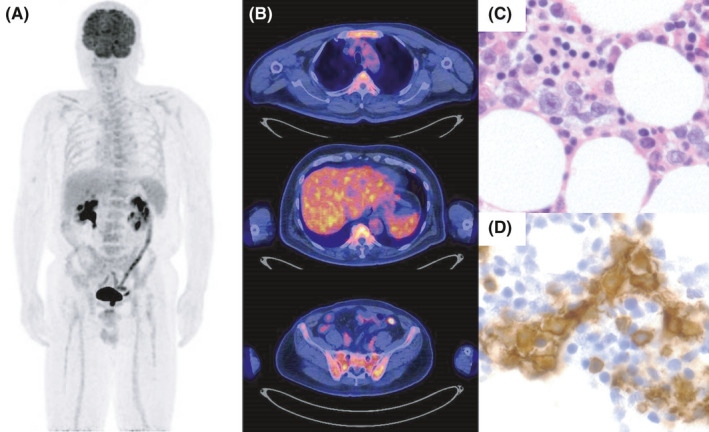
Intense uptake of 18‐fluorodeoxyglucose (FDG) disseminated throughout the bone marrow at initial diagnosis in the FDG‐PET maximum intensity projection mode (MIP) (A) Uptake by the bone marrow at the sternum, vertebrae and pelvis (SUV max, 6.77; Deauville five‐point scale: 4) (B) Histopathology of the bone marrow biopsy showing proliferation of large lymphoid cells; immunohistochemical staining showing HE (C) and CD20‐staining (D) (magnification, ×400)

**FIGURE 2:**
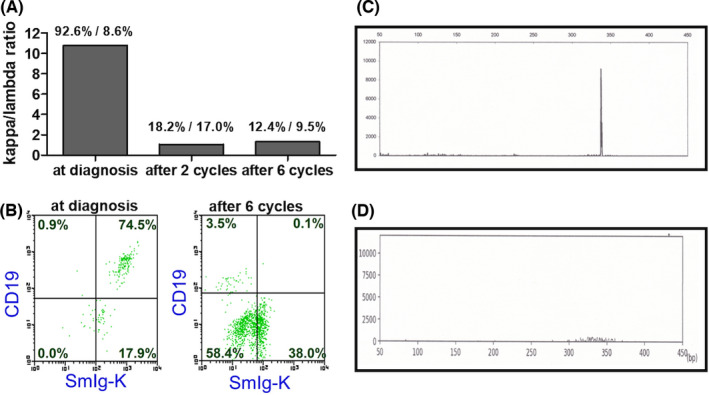
Kappa/lambda ratio calculated by percentage of surface immunoglobulin kappa or lambda positive cells using flowcytometric analysis (A) Flowcytometric analysis. Lymphoma cells with CD19 and surface immunoglobulin kappa double positive at diagnosis and after six cycles of Rituximab‐based CHOP (B) IGH rearrangement analysis showing positive clonality peak at VH(FR1)/JH region at the time of diagnosis (C) and disappearance of clonality peak after two cycles of Rituximab‐based CHOP therapy (D)

**FIGURE 3:**
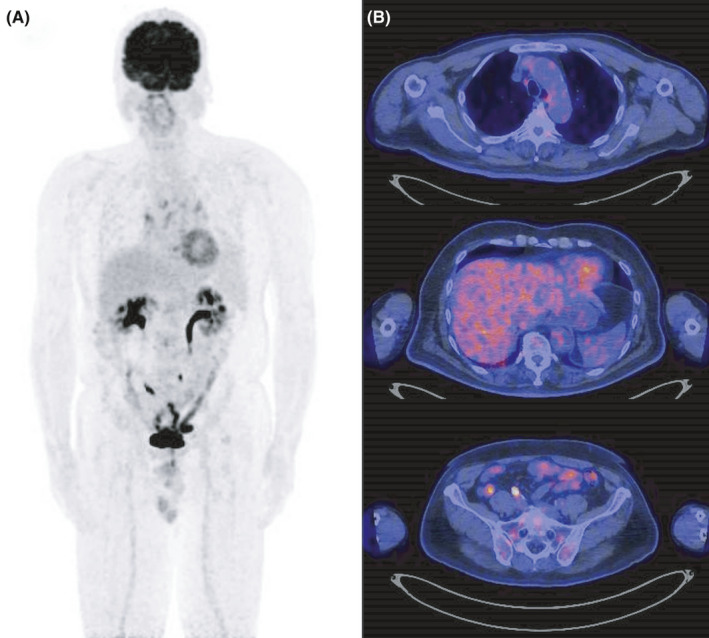
Fluorodeoxyglucose uptake by bone marrow after six cycles of Rituximab‐based CHOP therapy by MIP imaging (A) Uptake by bone marrow of the sternum, vertebrae and pelvis, equal or lower than by the mediastinum (Deauville five‐point scale: 2) (B)

We report a 64‐year‐old man admitted with fever of several weeks´ duration. Infection and collagen disease were excluded, but no diagnosis could be made. Computer tomography revealed no lymphadenopathy or extra‐nodal tumor but 18‐fluorodeoxyglucose‐positron emission tomography (18^F^ FDG–PET) revealed increased uptake disseminated through the BM (SUV max 6.77, Figure 1A,B). Iliac crest BM biopsy showed large CD20^+^ B‐cell infiltration, consistent with B‐cell neoplasia (Figure 1C,D). CD20^+^ lymphoid cells were positive for CD5, bcl‐2, and MUM‐1 but negative for CD10, bcl‐6, and EBER‐1 in immunohistochemistory (IHC). Furthermore, FCM of BM aspirates showed increased CD19^+^ and CD20^+^ B‐cell populations and marked kappa light chain restriction with 92.6% kappa and only 8.6% lambda was also observed in single color analysis (Figure 2A). Lymphoma cells expressing CD19 and surface immunoglobulin kappa were also increased with 74.5% (Figure 2B). Clonal immunoglobulin heavy chain (IGH) gene rearrangement was also detected by the polymerase chain reaction (PCR; Figure 2C). Intravascular large B‐cell lymphoma was suspected but excluded by random skin biopsy. Taken together, these data led to a diagnosis of PBML. The Hans algorithm is classified in non‐germinal center B‐cell type by IHC expression.^5^ The International Prognostic Index was classified as high‐intermediate risk with 3‐points (age, clinical stage, and LDH elevation). R‐CHOP therapy was initiated. Soon thereafter, fever completely resolved. Soluble IL‐2 receptor (sIL‐2R) level was drastically decreased from 4005 U/ml (normal range: 157–474) at diagnosis to 656 U/ml after first cycle of R‐CHOP. Interim evaluation of BM aspirates after two cycles of R‐CHOP showed a normal ratio of kappa to lambda with 18.2%–17.0% (Figure 2A). IGH gene rearrangement abnormalities disappeared completely (Figure 2D), suggesting an early major response to R‐CHOP therapy. After completion of six cycles of R‐CHOP therapy, FDG‐PET and BM examination were repeated, confirming a metabolic complete response (CR; Figure 3A,B) with maintained normal kappa/lambda ratio, disappearance of CD19 and surface immunoglobulin kappa double‐positive lymphoma cells and lack of genetic abnormality (Figure 2A,B). sIL‐2R level was further decreased within normal range after four cycles of R‐CHOP and never increased.

## 3 DISCUSSION

In this report, we have described a case of PBML with an early response confirmed by FCM and IGH gene rearrangement analysis. Thus, early chemosensitivity assessment could have predictive value for CR or PFS^6^, ^7^ and avoid unnecessarily aggressive treatments. Genetic assays of IGH rearrangement have previously been employed for detecting minimal residual disease in B‐cell leukemia.^8^ Unlike common DLBCL forming solid tumors, PBML is a special subtype of DLBCL which can be repeatedly sampled and evaluated by FCM and IGH rearrangement, as with leukemia. Early disappearance of IGH abnormalities could be a promising biomarker for major response to chemotherapy in PBML. Furthermore, repetitive evaluation for IGH abnormalities after treatment would be useful for guaranteed complete response or detection of early relapse.

## 4 CONCLUSION

Primary bone marrow B‐cell lymphoma is a rare DLBCL entity with potentially poor prognosis but conventional R‐CHOP therapy could be curative. To establish guidelines for standard therapies and to robustly predict prognosis, data on more cases are needed. Evaluation of early response by FCM and IGH assessments could be valuable for predicting treatment outcome and to optimize treatment intensity.

## CONFLICT OF INTEREST

The authors have no conflict of interest.

## AUTHOR CONTRIBUTIONS

YN and SK collected data and drafted the manuscript. YN, SK, YK, and AW were physicians providing chemotherapy. All authors reviewed the manuscript. TS supervised the study.

## ETHICAL STATEMENT

This article does not contain any studies with human participants performed by any of the authors.

## CONSENT

The written informed consent was obtained from the patient for publication.

## Data Availability

Research data are not shared.
